# Strong Activation of *ID1*, *ID2*, and *ID3* Genes Is Coupled with the Formation of Vasculogenic Mimicry Phenotype in Melanoma Cells

**DOI:** 10.3390/ijms25179291

**Published:** 2024-08-27

**Authors:** Nickolai A. Tchurikov, Amalia A. Vartanian, Elena S. Klushevskaya, Ildar R. Alembekov, Antonina N. Kretova, Viktoriya N. Lukicheva, Vladimir R. Chechetkin, Galina I. Kravatskaya, Vyacheslav S. Kosorukov, Yuri V. Kravatsky

**Affiliations:** 1 Department of Epigenetic Mechanisms of Gene Expression Regulation, Engelhardt Institute of Molecular Biology Russian Academy of Sciences, 119334 Moscow, Russia; giedre@inbox.ru (E.S.K.); alembeki@gmail.com (I.R.A.); tonya_kretova@mail.ru (A.N.K.); svetlaya.viktoriya@list.ru (V.N.L.); vladimir_chechet@mail.ru (V.R.C.); galina.kravatskaya@gmail.com (G.I.K.); jiri@eimb.ru (Y.V.K.); 2 Department of Experimental Diagnosis and Therapy of Tumors, N.N. Blokhin National Medical Research Center of Oncology of the Ministry of Health of Russia, 115478 Moscow, Russia; zhivotov57@mail.ru (A.A.V.); atgtga@mail.ru (V.S.K.); 3 Center for Precision Genome Editing and Genetic Technologies for Biomedicine, Engelhardt Institute of Molecular Biology Russian Academy of Sciences, 119334 Moscow, Russia

**Keywords:** co-expressing genes, melanoma, vasculogenic mimicry, Matrigel, RNA-Seq, *ID1*, *ID2*, *ID3*, rDNA

## Abstract

Gene expression patterns are very sensitive to external influences and are reflected in phenotypic changes. It was previously described that transferring melanoma cells from a plastic surface to Matrigel led to formation of de novo vascular networks—vasculogenic mimicry—that are characteristic to a stemness phenotype in aggressive tumors. Up to now there was no detailed data about the gene signature accompanying this process. Here, we show that this transfer shortly led to extremely strong epigenetic changes in gene expression in the melanoma cells. We observed that on Matrigel numerous genes controlling ribosome biogenesis were upregulated. However, most of the activated genes were inhibitors of the differentiation genes (*ID1*, *ID2*, and *ID3*). At the same time, the genes that control differentiation were downregulated. Both the upregulated and the downregulated genes are simultaneously targeted by different transcription factors shaping sets of co-expressed genes. The specific group of downregulated genes shaping contacts with rDNA genes are also associated with the H3K27me3 mark and with numerous lincRNAs and miRNAs. We conclude that the stemness phenotype of melanoma cells is due to the downregulation of developmental genes and formation of dedifferentiated cells.

## 1. Introduction

Epigenetic changes in gene expression lead to changes in phenotypes of cells. The changes are induced by different endogenous and exogenous factors and are due to changes in transcriptomic signatures and in corresponding cellular processes. The study of underlying molecular mechanisms is of great importance for understanding normal development and different pathologies. Cancer cells are of great interest in this respect. Melanoma, the most aggressive type of skin cancer, originates from melanocytes. Treating melanoma is a challenge due to drug resistances and dissemination of melanoma cells [1]. One resistance problem arises from the propensity of melanoma cells to develop vasculogenic mimicry through their capacity to build vascular channels, cords, and sinuses that lack endothelial cells and are formed by melanoma cells together with endothelial cells from normal angiogenesis [2,3]. It has been proposed that melanoma cells acquire a stem-cell-like transendothelial phenotype over a course of hypoxia [4]. The pattern of melanoma cells in tumors resembles a honeycomb structure [5]. The vasculogenic mimicry phenotype is associated with stemness and aggressive behavior in cancer cells [6].

It is known that changes in phenotype of normal and cancer cells are tightly linked with regulation of gene expression from both endogenous and exogenous factors [7]. As a result, each cell type is formed by the expression of specific sets of genes [8]. Dynamic changes in development and maintenance of differentiated states are determined by different epigenetic mechanisms, including DNA methylation, histone modifications, non-coding RNAs, and higher-order 3D chromosome structures [9,10,11,12]. Cancers may be caused by epigenetic dysregulations or by exogenous factors. At the same time, cancer induces epigenetic changes in cells [13]. In all cancers, there are epigenetic changes driving cancer phenotypes [14].

The aim of this study was to uncover the changes in the whole-genome gene expression patterns of the same melanoma cells that, in different growth conditions, either did not form or did form capillary-like structures. It was previously described that the vasculogenic mimicry phenotype of melanoma cells is associated with the formation of 3D capillary-like structures by aggressive melanoma cells growing on Matrigel [15]. It is known that the same cells growing in monolayers in 2D or in 3D have differences in the central energy metabolism of the cancer cells [16]. Matrigel is used as an extracellular matrix for 3D cell cultures and is known to alter cellular phenotypes and gene expressions in cancer cells that exhibit the vasculogenic mimicry phenotype [17]. 

Here, we performed transcriptome analysis of Mel Z cells cultivated either in 2D on a plastic surface or in 3D on Matrigel. The cells were isolated in the N.N. Blokhin National Medical Research Center of Oncology of the Ministry of Health of Russia from a metastasis into a lymph node in a patient with disseminated melanoma [18]. It is known that a 3D culture more closely represents a tumor tissue microenvironment and cell-to-cell interactions. This is true for the melanoma cells that form clusters and capillary-like structures on Matrigel [18,19]. Our transcriptome analysis revealed dramatic epigenetic changes in the expression patterns of the Mel Z melanoma cells grown in the same medium after being transferred from a plastic surface to Matrigel. We detected that the Mel Z cells cultivated on Matrigel that shaped capillary-like structures demonstrated high activation of the inhibitors of the differentiation genes (*ID1*, *ID2*, and *ID3*). We found that both the upregulated and the downregulated genes were simultaneously regulated by numerous transcription factors and included genes shaping contacts with rDNA genes. The downregulated genes contacting with rDNA were highly associated with the H3K27me3 mark and numerous lincRNAs and miRNAs. 

## 2. Materials and Methods

### 2.1. Reagents

RPMI-1640 medium, glutamine, and gentamicin sulfate were purchased from PanEco (Moscow, Russia). Fetal bovine serum (FBS) was purchased from Hyclone (South Logan, UT, USA). Matrigel was purchased from BD Bioscience (Franklin Lakes, NJ, USA). Trizol reagent and PureLink RNA Micro Kit were purchased from Invitrogen (Carlsbad, CA, USA). RNA 6000 Nano Kit was purchased from Agilent (Santa Clara, CA, USA). Dynabeads^®^ mRNA Purification Kit was purchased from Ambion (Austin, TX, USA). NEBNext^®^ Ultra™II RNA Library Prep Kit for Illumina was purchased from NEB (Ipswich, MA, USA). 

### 2.2. Cell Culture

The derivation and source of the Mel Z melanoma cell line have been reported previously [18]. The cells were obtained from the N.N. Blokhin National Medical Research Center of Oncology of the Ministry of Health of Russia. The cells were initially propagated on a plastic surface in RPMI-1640 medium supplemented with 10% fetal calf serum, 2 mM glutamine, and 0.1% gentamicin sulfate at 37 °C in a humidified atmosphere containing 5% CO_2_ and up to 10 million cells with 70–75% confluency. Then the cells were divided into three equal samples and seeded either on plastic or on Matrigel. Growth took place under identical conditions for 20 h; then the cells were used for RNA isolation. Petri dishes coated with Matrigel (BD Bioscience, USA) were prepared as follows: Matrigel (8.7 mg/mL) was thawed at 4 °C, and 6 mL was quickly added to each 10 cm dish and allowed to solidify for 30 min at room temperature; then it was placed in a humidified 5% CO_2_ incubator for one hour at 37 °C. 

### 2.3. RNA Isolation and RNA-Seq Procedure

The melanoma Mel Z cells grown under different conditions were uniformly and separately lysed with Trizol reagent (Invitrogen). Total RNA was extracted from cells lysed with Trizol using a PureLink RNA Micro Kit (Invitrogen) in accordance with the manufacturer’s instructions. RNA quality was checked using a Bioanalyzer and RNA 6000 Nano Kit (Agilent). Poly(A)+ RNA was purified using a Dynabeads^®^ mRNA Purification Kit (Ambion). The Illumina library was prepared from poly(A)+RNA with a NEBNext^®^ Ultra™II RNA Library Prep Kit for Illumina^®^ (NEB), according to the manual. Sequencing was performed on a 50 bp read length. At least 10 million reads were generated for each sample. RNA-Seq data were deposited in the Gene Expression Omnibus (GEO) repository under accession number GSE221877.

### 2.4. RNA-Seq Analysis

To ensure the consistency of the genome and annotation, we employed the same version of the genome build for both sequence and annotation (*H. sapiens* hg38/GRCh38 Ensembl release 106 build, http://ftp.ensembl.org/pub/release-106/, accessed on 8 August 2024). Both RNA-Seq experiments were analyzed uniformly using the following method: As the first step, all reads were processed using Trimmomatic v. 0.39 [20] to remove short reads (<20 bp) and low-quality ends (Q < 22) and to maintain acceptable read quality throughout all read lengths. The following options were used: LEADING: 22, TRAILING: 22, SLIDINGWINDOW: 4:26, MINLEN: 20. Trimmed reads were accurately quantified to *H. sapiens* Ensembl v.106 genome/annotation by RSEM 1.3.1 [21] using the following options: --fragment-length-mean 255 --star --calc-ci --ci-memory 30720 for both replicates separately. The results were averaged between replicates using an in-house R script. To ensure full consistency with the RSEM results, we mapped the trimmed reads using STAR aligner 2.7.5c [22] with the same options as for RSEM (--outSAMunmapped Within --outFilterType BySJout --outSAMattributes NH HI AS NM MD --outFilterMultimapNmax 20 --outFilterMismatchNmax 999 --outFilterMismatchNoverLmax 0.04 --alignIntronMin 20 --alignIntronMax 1000000 --alignMatesGapMax 1000000 --alignSJoverhangMin 8 --alignSJDBoverhangMin 1 --sjdbScore 1 --quantMode TranscriptomeSAM). We applied featureCounts 2.0.3 [23] with the recommended options (-t exon -g gene_id) to assign the alignments produced by STAR to genome features. Then, we applied the DESeq2 [24] R library to obtain differential expression tables for the pairs MP-MM and MM-MMI. In addition, DESeq2 was applied to obtain a scatterplot of quantified datasets for consistency control between replicates. An in-house Perl script was applied to assign ISO gene names to gene IDs for further genetic and GO analyses. 

A consistency check between replicates was performed using deepTools [25]. Pearson and Spearman correlation coefficients for the MP (*r* = 0.99, *ρ* = 0.76) and MM (*r* = 0.99, *ρ* = 0.69) datasets confirmed the high degree of consistency and thus the reliability of the obtained RNA-Seq data. Scatterplots of both raw and quantified data are shown in Figure S1.

### 2.5. Statistical Assessment

Gene differential expression *p*-values, calculated by DESeq2 [24], were adjusted by FDR/Benjamini–Hochberg method for multiple testing to reduce the number of false positives. Genes with FDR > 0.05 were omitted from further analyses. 

Overlapping *p*-values of gene lists were assessed using the exact test of multiset intersections from the SuperExactTest [26] R library that permits analysis of relationships among multiple gene sets that is otherwise impossible through the traditional pair-wise Fisher’s exact test. The *p*-values calculated by SuperExactTest were adjusted by Bonferroni method for multiple testing.

The *p*-values calculated by Enrichr (https://maayanlab.cloud/Enrichr/enrich, accessed on 8 August 2024) were adjusted according to FDR (false discovery rate) corrections by Benjamini–Hochberg method. All scripts mentioned in the manuscript were uploaded to Github: https://github.com/lokapal/Melanoma (accessed on 8 August 2024). 

## 3. Results

### 3.1. Transferring Melanoma Cells from a Plastic Surface to Matrigel Dramatically Changed the Expression of about 2700 Genes 

**Figure 1:**
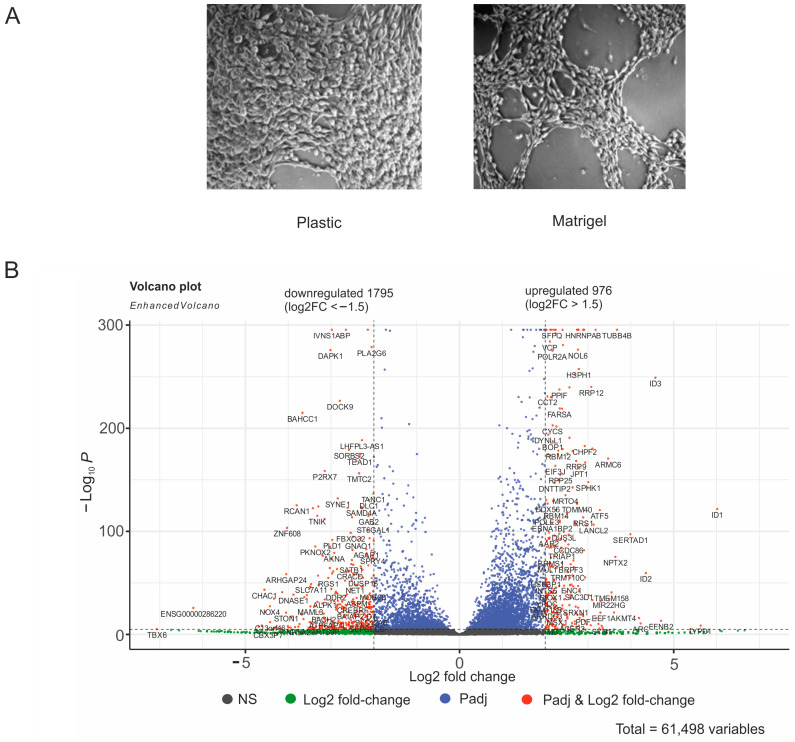
Effects of growth conditions on Mel Z cells. (**A**) Melanoma cells grown for 20 h on plastic or on Matrigel. (**B**) Differential expression of genes in melanoma cells cultured on plastic (MP) or on Matrigel (MM). The volcano plot presents statistically significant log_2_ fold changes in expression determined by DESeq2 [16] in RNA-Seq experiments. The *p*-values were corrected by Benjamini–Hochberg method for multiple testing that is built-in in DESeq2. Upon transfer of cells to Matrigel and incubation for 20 h in the same medium, 976 genes were upregulated and 1795 were downregulated under log_2_FC < ±1.5 and FDR ≤ 0.05. The complete list of 10,221 transcribing genes and their expression values on plastic and Matrigel are shown in Table S1.

We expected to observe changes in gene expression induced by transferring cells from plastic to Matrigel and allowing subsequent growth for 20 h (see Methods). This transfer and rather short incubation are sufficient to allow cells to develop 3D capillary-like structures [18,19,27]. The phenotypes of the melanoma Mel Z cells grown on plastic and Matrigel are shown in Figure 1A. The monolayer on the plastic surface appeared to consist of spindle-shaped or multipolar cells. On Matrigel, the cells formed capillary-like structures, which were associated with the cells acquiring an elongated shape. Clearly, this phenotype was formed under the control of a specific set of genes and factors. To identify the genes that are characteristic of the Mel Z cells that possessed stemness, we performed differential expression analysis. 

Figure 1B shows that in our experiments the transfer to Matrigel induced changes, by at least 2.8-fold (log_2_ FC ≥ 1.5), in the expression of about 2700 genes. To shape the vasculogenic mimicry phenotype, the Mel Z cells that were transferred from plastic to Matrigel needed to upregulate 976 genes and downregulate 1795 genes. The complete list of genes and their expression levels in the cells grown on the plastic surface (melanoma–plastic (MP)) and on Matrigel (melanoma–Matrigel (MM)) are shown in Table S1. Such dramatic changes in expression were probably due to the up to 64-fold activation of *ID1*, *ID2*, and *ID3* genes during growth on Matrigel. The FDR values for *ID* genes activation (2.21 × 10^−120^ for *ID1*, 8.14 × 10^−59^ for *ID2*, 3.46 × 10^−247^ for *ID3*) are ⋘ 10^−6^, that ensures that these results were not obtained by chance. The genes specify helix–loop–helix (HLH) proteins that can form heterodimers with members of the basic HLH family of transcription factors. Interestingly, *ID1*, *ID2*, and *ID3* genes encode proteins that lack a basic DNA-binding domain and can therefore inhibit DNA binding of the HLH proteins with which they interact. This should inhibit the DNA binding of different transcription factors and change the expression of numerous genes controlling cell growth and differentiation [27,28]. We hypothesize that the extreme activation of these *ID* genes, which are considered dominant negative regulators of other HLH transcription factors and inhibitors of differentiation [29,30], could lead to downregulation of many genes. 

**Figure 2:**
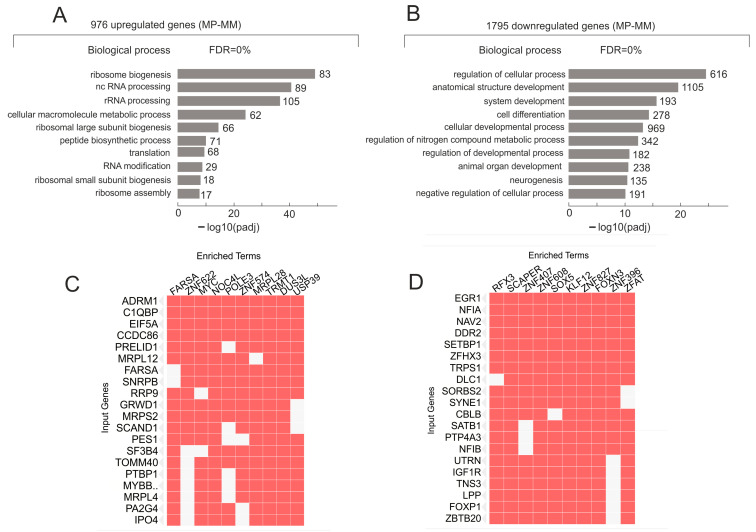
Characterization of genes upregulated or downregulated after transfer of melanoma cells to Matrigel. (**A**) Top 10 Gene Ontology (GO) biological process associations of 976 upregulated genes using Profiler (https://biit.cs.ut.ee/gprofiler/gost, accessed on 8 August 2024). Values to the right of bars show the number of genes associated with a process. The complete list of corresponding genes is shown in Table S2. (**B**) Top 10 GO biological process associations of 1795 downregulated genes using Profiler. Values to the right of bars show the number of genes associated with a process. The complete list of corresponding genes is shown in Table S3. (**C**) Genes upregulated on Matrigel (“input genes”) are co-expressed with different transcription factors (indicated as “Enriched Terms”) across different human cell lines and tissues. Top 20 input genes are also shown. The search was performed in https://maayanlab.cloud/Enrichr/enrich# (accessed on 8 August 2024) for ARCHS4 TFs Coexp. The complete list of 219 sets of co-expressing genes and corresponding transcription factors is shown in Table S4. (**D**) Genes downregulated on Matrigel (“input genes”) are simultaneously regulated by different transcription factors. Data were obtained by a search of Enrichr Submissions TF-Gene Cooccurrence (https://maayanlab.cloud/Enrichr/enrich, accessed on 8 August 2024). The complete list of 457 sets of co-regulated genes and corresponding transcription factors is shown in Table S5.

To understand the biological functions associated with the genes that were up- or downregulated in the melanoma cells after the transfer to Matrigel, we performed a Gene Ontology (GO) search. Figure 2A shows the top ten GO biological process items for upregulated genes that were involved in development of vasculogenic mimicry phenotypes by the Mel Z cells on Matrigel. Most of these process items are associated with ribosome biogenesis, translation, and nc RNA processing. The data suggest that the development of stemness phenotypes requires the activation of biogenesis and ribosome activity. The result is consistent with the data on the linkage of nucleoli function with cancer genesis [31,32].

At the same time, about 1800 genes were downregulated during this process; among them were genes that are involved in the regulation of development and differentiation (Figure 2B). Paradoxically, it follows that the development of capillary-like structures by the melanoma cells was associated with the downregulation of about 1800 genes controlling the development of the anatomical structure of genes and the cellular developmental processes (Figure 2B). We suppose that these data indicate that the formation of vascular channels, cords, and sinuses by melanoma cells in 3D cultivation is a result of dedifferentiation. The view is consistent with data suggesting that vascular channels shaped by melanoma cells can mimic the embryonic vascular network pattern [28]. The supposition is also consistent with observations that the most aggressive tumors are shaped by nondifferentiated cells [27,33]. The conclusion that the melanoma cells transferred from plastic to Matrigel underwent dedifferentiation is independently supported by our observation that *ID1*, *ID2*, and *ID3* genes (Figure 1B), which can inhibit numerous transcription factors from the HLH family, important regulators of cellular growth and differentiation [28,29,30], were highly activated (Figure 1B).

### 3.2. Upregulated and Downregulated Genes Are Simultaneously Regulated by Many Transcription Factors

To understand the mechanisms that led to the upregulation of about 900 genes we used a search of the Enrichr databases for ARCHS4 TFs Coexp, https://maayanlab.cloud/Enrichr/enrich#, and detected an extremely strong association of these genes with different transcription factors (*p*_adj_ ≤ 3 × 10^−47^). The database includes genes that are co-expressed with different transcription factors across different human cell lines and tissues [34]. Ten of these transcription factors are indicated as “Enriched Terms” in Figure 2C. The top 20 upregulated genes are also shown in Figure 2C (‘input genes”). Table S4 shows the complete list of 219 sets of co-expressions in different combinations of the upregulated genes (from 94 to 25 genes in a set). Among them there are also 23 transcription factors regulating these 976 upregulated genes. The co-expression combinations of the upregulated genes indicate that growth on Matrigel led to activation of 23 transcription factors regulating these sets of genes.

The downregulated genes also are co-regulated by numerous transcription factors (Figure 2D). Data were obtained by a search of Enrichr Submissions TF-Gene Cooccurrence (https://maayanlab.cloud/Enrichr/enrich, accessed on 8 August 2024). There are 457 sets of the downregulated genes simultaneously regulated by different transcription factors (Table S5). The sets possess from 88 to 25 downregulated genes (*p*_adj_ ≤ 3 × 10^−21^). Among them there are also 101 transcription factors regulating these 1795 downregulated genes. The co-regulation combinations of the downregulated genes indicate that growth on Matrigel led to repression of 101 genes encoding transcription factors regulating these sets of genes. Among these 101 genes there are 31 ZNF genes. Taken together with the data on the activation of 23 genes specifying transcription factors regulating the upregulated genes, the data suggest that growth on Matrigel induces both upregulation and downregulation of numerous transcription factors. As a result, big sets of genes are activated or repressed. 

The data strongly indicate that upon cultivation of Mel Z cells on Matrigel for 20 h dramatic changes in expression occurred for genes that have a complex regulation. The mechanisms of this type of regulation are not clear yet. We mentioned that the downregulated genes, which mainly correspond to developmental genes, include 58 ZNF genes, while the upregulated genes that are enriched with genes involved in ribosome biogenesis, have 16 ZNF genes. The sets of co-expressing downregulated genes often have clusters of ZNF genes, while the sets of co-expressing upregulated genes mostly lack these genes (Tables S4 and S5).

### 3.3. Relationship of Upregulated and Downregulated Genes with rDNA-Contacting Genes and Genes with Hot Spots of DSBs in the Human Genome 

To understand further the molecular mechanisms behind the upregulation or the downregulation of genes during growth of the melanoma cells for 20 h on Matrigel, we attempted to use an independent approach. It has been reported that rDNA clusters swiftly shape dynamic contacts with key genes that are involved in differentiation and cancer, and that DSBs hotspots often occur in these genes [32,35,36,37]. 

We decided to explore whether the up- and downregulated genes detected in this study overlapped with a set of genes generated independently using a different procedure by performing 4C-rDNA experiments and genome-wide mapping of hot spots of DSBs [36]. We used the available data on HEK293T cells, which, similar to melanoma cells, have a common origin from neural crest cells. Neurons, glia, and melanocytes are originated from the neural crest [38,39,40] that develops into most of the peripheral nervous system and into some non-neural cell types, including pigment cells in the skin. 

**Figure 3:**
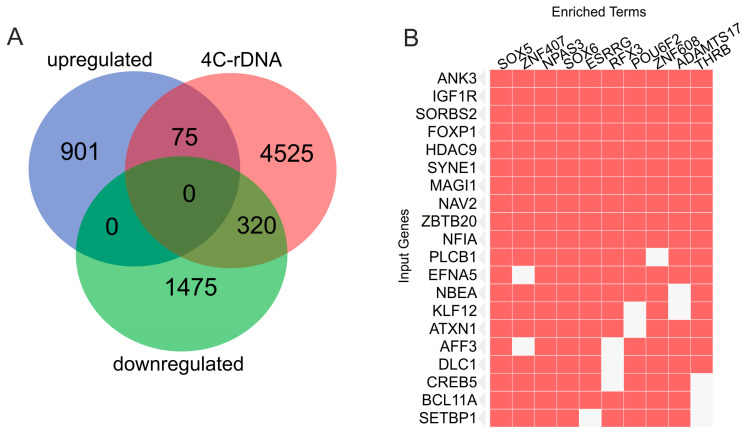
Relationships between the upregulated and downregulated genes induced by growth on Matrigel and rDNA-contacting genes. (**A**) Venn diagram showing intersections with rDNA-contacting genes (4C-rDNA). (**B**) The 320 downregulated genes contacting with rDNA are simultaneously regulated by different transcription factors (“enriched terms”). The top 20 genes are shown (“input genes”). Data were obtained by a search of corresponding genes in Enrichr Submissions TF-Gene Co-occurrence (https://maayanlab.cloud/Enrichr/enrich#, accessed on 8 August 2024).

Between the downregulated genes in the melanoma cells incubated on Matrigel and rDNA-contacting genes (4C-rDNA), we detected 320 overlapping genes (Figure 3A). The complete list of overlapping genes is given in Table S6. SuperExactTest (see Methods) shows that this overlapping of independently generated gene lists using completely different experimental procedures (4C-rDNA and RNA-Seq) is non-random (*p*-value < 2.9 × 10^−57^). These 320 genes, as expected from the previous data (Figure 2D), are simultaneously regulated by different transcription factors (Figure 3B). The similar search for the 75 upregulated genes revealed only a few associated TFs).

In order to understand the possible mechanisms behind the silencing of these genes we used a search in Enrichr database ENCODE Histone Modifications 2015 (https://maayanlab.cloud/Enrichr/enrich#, accessed on 8 August 2024) and found association of these 320 rDNA-contacting genes with the H3K27me3 mark in ten different human cells and tissues (Table S7). Mostly, each of these genes is silenced by this mark in different cell types. It follows that downregulation of this set of genes was due to deposition of the H3K27me3 mark. Interestingly, the remaining 1475 downregulated genes (Figure 3A) exhibit no association with this mark. That is why we suppose that rDNA clusters by some way could be involved in the deposition of this mark to the set of rDNA-contacting genes. 

Similarly, a search in Enrichr database (TargetScan microRNA 2017) revealed a high association (up to *p*-value < 3.6 × 10^−14^) of sets of these genes in different combinations with 211 human miRNAs (Table S8). The data indicate that one possible mechanism of gene downregulation in the melanoma cells grown on Matrigel was the miRNA pathway acting in the cytoplasm. Again, the remaining 1475 downregulated genes (Figure 3A) exhibit no association with miRNAs in the Enrichr database. The lists of genes silenced by the H3K27me3 mark and by miRNAs overlap (Tables S7 and S8). It follows that some genes from the set of 320 overlapping genes in the melanoma cells were silenced on both transcriptional and translational levels.

**Figure 4:**
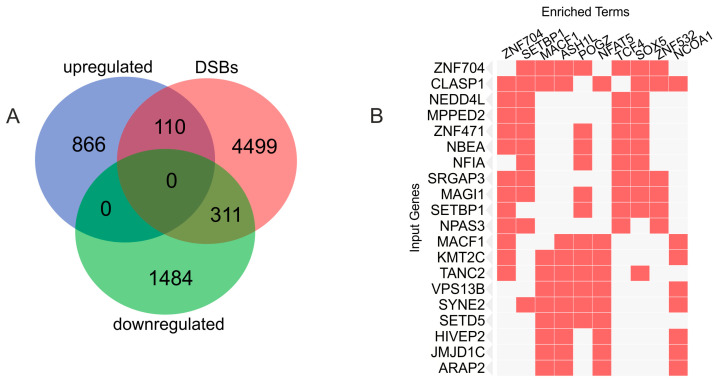
Relationships between the upregulated and downregulated genes induced by growth on Matrigel and genes possessing DSBs hot spots. (**A**) Venn diagram showing intersections with genes possessing DSBs hot spots (DSBs). (**B**) The 311 downregulated genes frequently possessing DSBs are simultaneously regulated by different transcription factors (“enriched terms”). The top 20 genes are shown (“input genes”). Data were obtained by a search of corresponding genes in Enrichr Submissions TF-Gene Cooccurrence (https://maayanlab.cloud/Enrichr/enrich#, accessed on 8 August 2024).

Independently, we found intersections between the genes changing expression in the melanoma cells during growth on Matrigel and the differentiation genes that possess frequent DSBs [37]. Figure 4A shows that there are 311 downregulated genes and 110 upregulated genes overlapping with genes with DSBs hotspots (Figure 4A). SuperExactTest (see Methods) shows that this overlapping of independently generated gene lists using completely different experimental procedures (mapping of DSBs and RNA-Seq) is non-random (*p*-value < 2.3 × 10^−52^ for 311 intersecting genes and *p*-value < 6.9 × 10^−6^ for 110 intersecting genes). The complete list of overlapping genes is given in Table S9. The data independently indicate that there were genes involved in differentiation among the upregulated and the downregulated genes. The 311 downregulated genes are co-regulated by different transcription factors (Figure 4B). The density of these factors is clearly lower than that for the 320 downregulated rDNA-contacting genes (Figure 3B). No associations for these 311 downregulated genes with miRNAs or the H3K27me3 mark were found. The similar search for the 110 upregulated genes revealed only a few associated TFs. 

The data indicate that the downregulated genes associated either with rDNA or with frequent DSB breakage are regulated differently. Although both groups of genes are regulated by numerous transcription factors, the set of genes associated with rDNA is silenced by multiple miRNAs and by deposition of the H3K27me3 mark, while the group of the downregulated genes associated with DSBs are regulated by some other pathways, which might be only by transcription factors, including factors repressing transcription. 

### 3.4. Association of lincRNAs and Active Histone Marks with Upregulation of 976 Genes in Melanoma Cells

**Figure 5:**
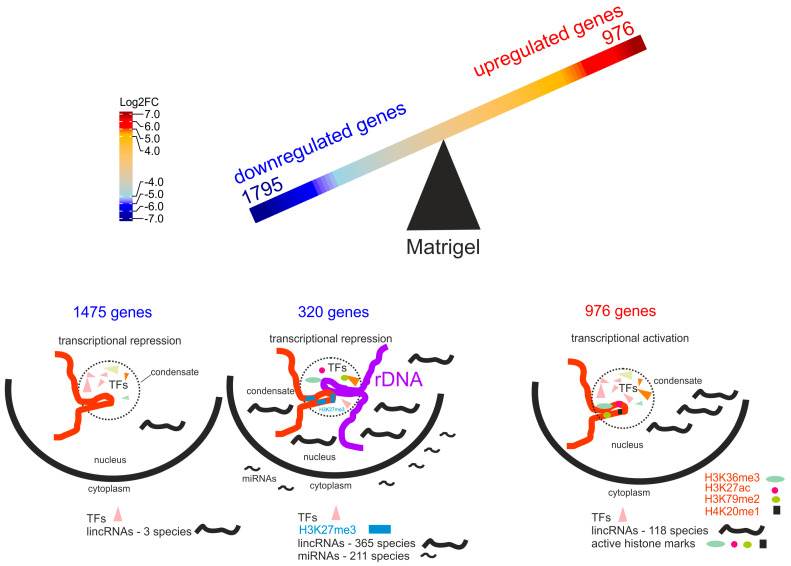
Schematic presentation of the mechanisms involved in downregulation or upregulation of genes in melanoma cells growing on Matrigel. Among 1795 downregulated genes there are 320 rDNA-contacting genes. Chromatin loops inside micro-condensates are shown.

The detection of a high association of 320 rDNA-contacting genes with numerous species of miRNAs prompted us to a search in the same database (Enrichr Submissions—TargetScan microRNA 2017) for the upregulated genes. However, no association was detected for the 976 upregulated genes. At the same time we found a high association (*p*-value ≤ 10^−20^) of 118 species of lincRNAs with different groups of the downregulated genes (Table S10). These small RNAs are longer than 200 nucleotides and include promoter- or enhancer-associated RNAs proximal to different genes [41]. We suppose that lincRNAs played a role in the upregulation of the 976 genes. The search in the Enrichr Submissions—ENCODE Histone Modifications 2015 revealed that these 976 upregulated genes also are highly associated with several active histone marks—H3K36me3, H3K27ac, H3K79me3, and H4K20me1—in big (up to 190 genes) overlapping groups of these genes in different human cells and tissues (Table S11). It follows that along with simultaneous regulation by different transcription factors, big sets of lincRNAs and several histone marks were associated with the upregulation of these 976 genes in the melanoma cells (Figure 5).

### 3.5. Association of lincRNAs with Downregulated Genes

Next, we attempted to search whether the 1795 downregulated genes also are associated with lincRNAs. We detected that among the downregulated genes there are 320 genes shaping contacts with rDNA genes; the most part of the downregulated genes (1475 genes) do not form these contacts. Surprisingly, we detected that these 1475 genes are associated only with 3 lincRNAs species, while the 320 rDNA-contacting genes are associated with 365 different lincRNAs species (Tables S12 and S13, respectively). There was no overlapping between these 3 and 365 lincRNA species. Taken together with the above data on miRNAs (Table S8), these results strongly indicate that the 320 downregulated rDNA-contacting genes, in contrast to the other 1475 downregulated genes, are regulated with numerous miRNAs and lincRNAs.

It is known that in human cells there are about 15,000 lincRNAs that are involved in regulation of gene expression [41]. Previously we found that the upregulated genes are associated with 118 lincRNAs (Table S10). Interestingly, there are no common lincRNAs between these sets of 365 and 118 lincRNAs. It follows that the upregulated and the downregulated genes in the melanoma cells are connected with two different classes of lincRNAs. One class is associated with the upregulation of numerous genes and possesses active histone marks, while another class is associated with downregulating genes and possesses repressive histone marks and is associated with different miRNAs (Figure 5).

## 4. Discussion

### 4.1. Matrigel Induces an Epigenetic Switch in Melanoma Cells

Melanoma cells grown on a plastic surface are unable to form 3D structures, leading to an inadequate gene expression pattern compared to that of a tumor. For this reason we used Matrigel, which is an extracellular matrix membrane of organic origin containing collagen, laminin, fibronectin, and some other compounds that form a globular 3D network on polystyrene that matches, to some extent, the extracellular environment of cells in a tumor. In this study, we compared the expression of about 10,000 genes transcribed in melanoma cells cultivated on either plastic or Matrigel (Table S1). We expected to observe differences in expression but were surprised to find that Matrigel induced dramatic changes in more than 2700 genes, probably because it possesses strong signaling molecules. Of course, these changes are not solely related to the development of vasculogenic mimicry. Currently, it is believed that Matrigel represents the most adequate 3D experimental model [42], and we can therefore consider that the vasculogenic phenotype of the melanoma Mel Z cells on Matrigel that we observed (Figure 1A) and the detected expression patterns (Table S1) correspond only to some extent to the patterns in tumors in vivo. It follows that cultivating melanoma Mel Z cells on plastic strongly affects the gene expression and that the cells are very sensitive to changes in cultivation conditions. The changes in gene expression in the melanoma cells growing on Matrigel included the upregulation of 18 lincRNAs and the downregulation of 121 lincRNAs (Table S1), which have been shown to be important in the epigenetic regulation of key molecular processes, including histone modification and chromatin dynamics [43].

At the same time Matrigel induced bipolar changes in the expression of transcription factors: the activation of 23 transcription factors that are involved in co-regulation of the upregulated genes, and the repression of 101 transcription factors that are involved in co-regulation of the downregulated genes.

### 4.2. Are ID1, ID2, and ID3 Genes Responsible for the Vasculogenic Phenotype of Melanoma Cells?

We detected that the *ID1*, *ID2*, and *ID3* genes were the most activated (up to 64-fold) upon transfer to Matrigel (Figure 1). These genes inhibit DNA-binding of cognate helix–loop–helix proteins (HLH) by arresting their action and preventing their function as transcription factors. It may follow that this arrest prevents the development of a more differentiated state and induces the malignant phenotype. HLH proteins shape a large superfamily of transcriptional regulators (more than 100 in human cells) [44]. Some HLH proteins are cell-specific, and some are widely expressed. Among the latter is *MYC*, which is often affected in cancer cells [45]. HLH proteins control the cell cycle, cell lineage development, and tumorigenesis. We suppose that attacking the inhibitors of DNA-binding and differentiation [29,30] could prevent the stemness phenotype in melanoma cells. Recently, it was described that during induced erythroid differentiation of K562 cells, the *ID3* gene was the most downregulated, indicating that this inhibitor of differentiation should be switched off to allow for differentiation [46]. In this study, we detected high upregulation of three *ID* genes in melanoma cells shaping the stemness phenotype on Matrigel. The data suggest that the loss of differentiation is associated with the upregulation of *ID* genes in the cancer phenotype, while the more differentiated state is coupled with the downregulation of these genes. To study the direct causative relationship between the *ID* genes activation and vasculogenic phenotypes of Mel Z cells growing on Matrigel, we will study a possible effect of the downregulation of these genes on the development of vasculogenic phenotype (the data will be published separately).

### 4.3. Distinct Mechanisms in Regulation of Genes Changing Expression in Melanoma Cells Grown on Matrigel

The epigenetic switch that we observed in this study reflected the changes in expression of about 2700 genes developed during rather short time—20 h. The data illustrate both a strong dependence of eukaryotic cells on the environment and a swift response to the external changes. The only way to understand the mechanisms that are involved in the changes of expression of thousands genes is to search in different databases for associations of these genes with different processes. 

Using this approach we detected that growth of the melanoma cells on Matrigel led to the upregulation of about 1000 genes that are simultaneously regulated by three mechanisms. They include (i) the co-regulation by many different transcription factors, (ii) regulation by a set of 118 lincRNAs, and (iii) the deposition of several active histone marks (Figure 5). The latter includes the H3K27ac mark characteristic to active enhancers; the H336me3 mark, which is associated with transcription elongation; the H3K79me2 mark, which is linked with active transcription; and the H420me1 mark that occurs at transcriptionally active and, hence, more open chromatin regions. 

A major part of the downregulated genes is regulated by my many different transcription factors. The set of 320 downregulated genes that shape contacts with rDNA genes is co-regulated by many different transcription factors. At the same time we detected a strong association with 211 miRNA species and 320 rDNA-contacting downregulated genes (Table S8). These species are characteristic only to this set of genes (Figure 5). It is known that multiple small RNAs that are very similar to miRNAs (non-canonical miRNAs) are derived from rDNA transcripts [47,48]. But the set of these 211 miRNAs includes only canonical miRNAs (Table S8). At present we cannot explain a putative link between rDNA genes and this set of miRNAs.

We observed two big non-overlapping sets of 118 and 385 lincRNAs that are associated with the upregulated and the 320 downregulated genes, respectively. It is known that lincRNAs are concentrated in the chromatin and could play different roles there, from recruiting transcription factors to either activating or repressing gene expression to participating in the formation of 3D chromatin structures [41]. They include enhancer RNAs which could transcribe from both enhancers and surrounding sequences [49,50]. We detected that the most abundant set of lincRNAs is associated with the downregulated rDNA-contacting genes. The rest of the downregulated genes are associated only with 3 lincRNA species. That is why we suppose that lincRNAs could play a role in inter-chromosomal contacts of rDNA genes with different genomic regions where these 320 downregulated genes reside. Recently it was shown that rDNA-contacting genes are co-expressed with different lincRNAs [51]. Taken together these data independently support the idea of a role for lincRNAs in expression patterns of rDNA-contacting genes.

We suppose that multiple transcription factors could co-regulate groups of genes inside phase-separated condensates. Different chromatin loops, including loops with rDNA clusters, could be merged in micro-drops, each possessing many transcription factors and lincRNAs (Figure 5). Concentration of transcription factors and lincRNAs could be the source of the nucleation event leading to the formation of phase-separated condensates in each cell cycle. 

## Data Availability

RNA-Seq data were deposited in the Gene Expression Omnibus (GEO) repository under accession numbers GSE221876 and GSE221872.

## Supplementary Materials

The following supporting information can be downloaded at: https://www.mdpi.com/article/10.3390/ijms25179291/s1.

## Abbreviations

Mel Z, melanoma cell line; *ID1*, *ID2*, and *ID3* genes, inhibitors of the differentiation genes; lincRNAs, long intervening/intergenic noncoding RNAs; miRNAs, microRNAs containing 21 to 23 nucleotides; H3K27me3, histone H3 lysine 27 trimethylation mark; MP, melanoma cells grown on plastic; MM, melanoma cells grown on Matrigel; GO, Gene Ontology; HLH, helix–loop–helix transcription factors possessing the helix–loop–helix motif.
